# A simple real-time model for predicting acute kidney injury in hospitalized patients in the US: A descriptive modeling study

**DOI:** 10.1371/journal.pmed.1002861

**Published:** 2019-07-15

**Authors:** Michael Simonov, Ugochukwu Ugwuowo, Erica Moreira, Yu Yamamoto, Aditya Biswas, Melissa Martin, Jeffrey Testani, F. Perry Wilson

**Affiliations:** 1 Program of Applied Translational Research, Yale School of Medicine, New Haven, Connecticut, United States of America; 2 Joint Data Analytics Team, Yale School of Medicine, New Haven, Connecticut, United States of America; 3 Section of Cardiology, Yale School of Medicine, New Haven, Connecticut, United States of America; Royal Derby Hospital, UNITED KINGDOM

## Abstract

**Background:**

Acute kidney injury (AKI) is an adverse event that carries significant morbidity. Given that interventions after AKI occurrence have poor performance, there is substantial interest in prediction of AKI prior to its diagnosis. However, integration of real-time prognostic modeling into the electronic health record (EHR) has been challenging, as complex models increase the risk of error and complicate deployment. Our goal in this study was to create an implementable predictive model to accurately predict AKI in hospitalized patients and could be easily integrated within an existing EHR system.

**Methods and findings:**

We performed a retrospective analysis looking at data of 169,859 hospitalized adults admitted to one of three study hospitals in the United States (in New Haven and Bridgeport, Connecticut) from December 2012 to February 2016. Demographics, medical comorbidities, hospital procedures, medications, and laboratory data were used to develop a model to predict AKI within 24 hours of a given observation. Outcomes of AKI severity, requirement for renal replacement therapy, and mortality were also measured and predicted. Models were trained using discrete-time logistic regression in a subset of Hospital 1, internally validated in the remainder of Hospital 1, and externally validated in Hospital 2 and Hospital 3. Model performance was assessed via the area under the receiver-operator characteristic (ROC) curve (AUC). The training set cohort contained 60,701 patients, and the internal validation set contained 30,599 patients. External validation data sets contained 43,534 and 35,025 patients. Patients in the overall cohort were generally older (median age ranging from 61 to 68 across hospitals); 44%–49% were male, 16%–20% were black, and 23%–29% were admitted to surgical wards. In the training set and external validation set, 19.1% and 18.9% of patients, respectively, developed AKI. The full model, including all covariates, had good ability to predict imminent AKI for the validation set, sustained AKI, dialysis, and death with AUCs of 0.74 (95% CI 0.73–0.74), 0.77 (95% CI 0.76–0.78), 0.79 (95% CI 0.73–0.85), and 0.69 (95% CI 0.67–0.72), respectively. A simple model using only readily available, time-updated laboratory values had very similar predictive performance to the complete model. The main limitation of this study is that it is observational in nature; thus, we are unable to conclude a causal relationship between covariates and AKI and do not provide an optimal treatment strategy for those predicted to develop AKI.

**Conclusions:**

In this study, we observed that a simple model using readily available laboratory data could be developed to predict imminent AKI with good discrimination. This model may lend itself well to integration into the EHR without sacrificing the performance seen in more complex models.

## Abstract 2

Michael Simonov and colleagues present a model for predicting acute kidney disease using electronic data records.

## Author summary

**Why was this study done?:**

- Acute kidney injury (AKI) is an adverse event associated with significant morbidity and healthcare costs.
- Treatments for AKI are generally poor and largely supportive; thus, there has been a focus on early identification and prevention of AKI.
- There has been growing interest in harnessing electronic health data for the prediction of AKI in hospitalized patients.

**What did the researchers do and find?:**

- We developed a mathematical model that predicts which patients would develop AKI for 169,859 patients observed at three hospitals in the US (specifically in New Haven and Bridgeport, Connecticut) between 2012 and 2016.
- The model included several elements about the patients, including demographics, medical history, and bloodwork.
- The model was able to provide a good prediction for patients who would develop AKI and was also able to predict with fair discrimination whether the patient would need dialysis or whether they would die in the hospital.

**What do these findings mean?:**

- This model could be readily implemented on an electronic health record (EHR) to alert or caution healthcare providers of their patient’s risk for developing AKI and provide guided decision support to best help prevent this adverse event.
- In the future, there could be a study evaluating different interventions randomized to individual patients at high risk for developing AKI as per the model. This strategy could develop novel therapies for the prevention of AKI.

## Introduction

Among hospitalized patients, acute kidney injury (AKI) is strongly associated with increased costs, length of stay, and mortality [1, 2]. As such, hospital-acquired AKI is being evaluated as a potential quality measure by the Centers for Medicare and Medicaid Services [3]. AKI is diagnosed in relation to a rise in creatinine, but this marker rises late in the course of the syndrome [4, 5]. Real-time prediction of AKI prior to a creatinine increase holds promise to preempt such events through medication adjustment, avoiding nephrotoxins, optimizing hemodynamics, or engaging in other diagnostic or therapeutic procedures, including biomarker measurement [6].

Modern electronic health record (EHR) systems can provide readily accessible data (e.g., demographics, laboratory studies) to fuel scientific study and prediction modeling [7, 8]. There have been several attempts to utilize large medical data sets for predicting which patients will develop AKI; however, none are widely implemented in clinical settings [9–17]. Some of these models focus on AKI in the setting of cardiac intervention, e.g., percutaneous coronary intervention, or focus on specific populations such as children or the elderly and thus do not generalize to all hospitalized patients [11, 12, 18, 19]. Other studies are limited to intensive care unit (ICU) patients [14]. Three prior studies have leveraged usage of real-time data to predict AKI onset; however, no study has identified which clinical data elements provide the most “bang for the buck” for accurate predictions and ease of EHR implementation [10, 16, 17].

We set out to study the relationship between several sets of variables and imminent AKI onset to guide future research into AKI prediction and management. We wanted to compare the predictiveness of time-invariant (static) variables, such as sex and race, with time-varying (dynamic) variables, such as laboratory values and requirement for vasopressors. We hypothesized that time-varying data, such as lab studies, would be both effective to model AKI and would be sufficiently simple to facilitate rapid deployment into the EHR.

## Materials and methods

### Patients

**Fig 1:**
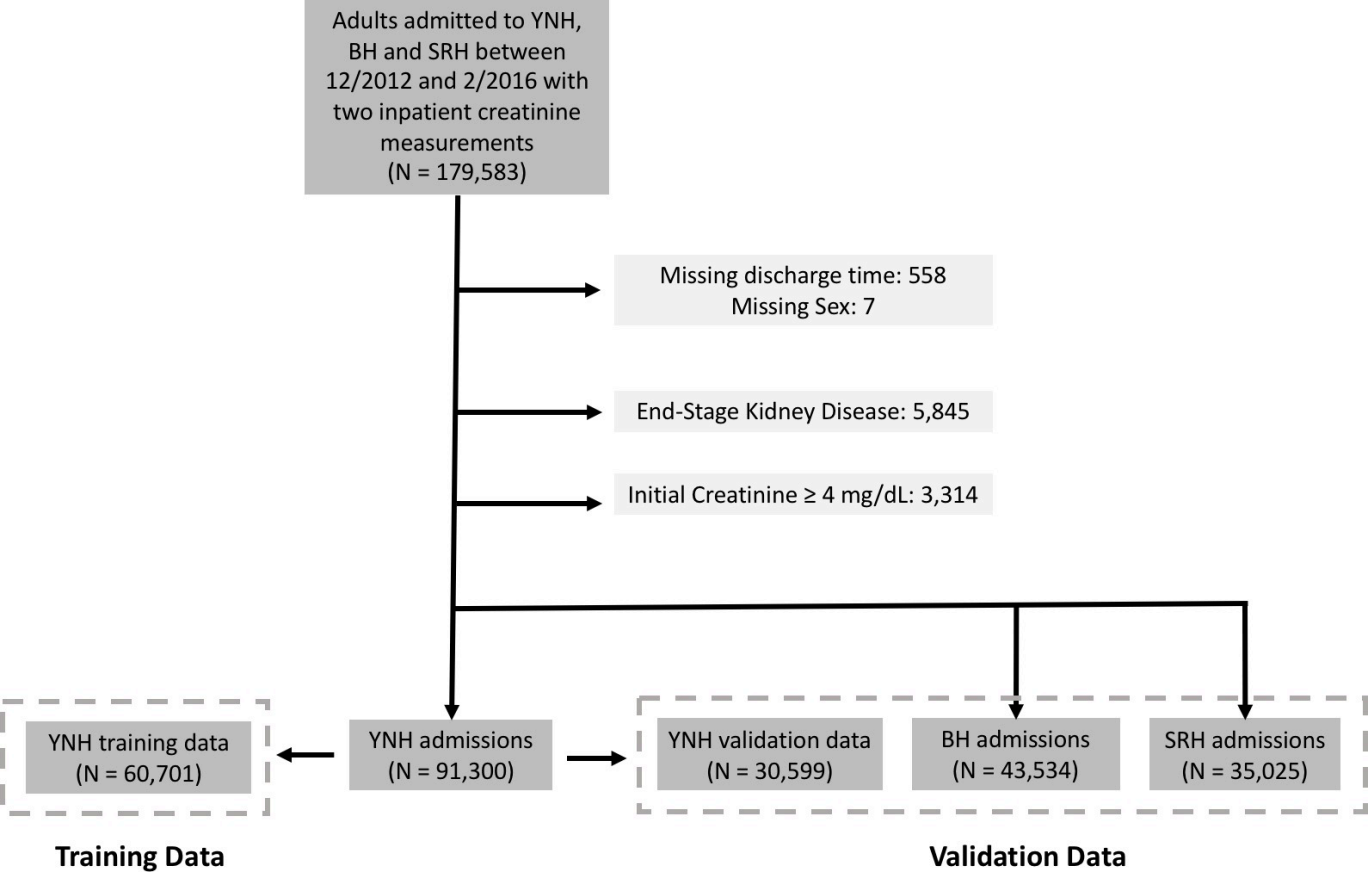
Flow diagram of the patient cohort with distribution of data among training and validation data sets.

This retrospective analysis evaluated data from hospitalized adult patients from three hospitals—Yale New Haven Hospital (YNHH), St. Raphael’s Hospital (SRH), and Bridgeport Hospital (BH)—admitted from 12/31/2012 to 2/09/2016 who had at least two inpatient creatinine values who were considered for inclusion. YNHH is a large tertiary care center set in an urban setting, SRH is a community teaching hospital, and BH is a private acute care hospital in an urban setting. All three hospitals used the same inpatient EHR (Epic, Verona, Wisconsin). Patients who were missing discharge times and those with an admission or prior ICD-9 or ICD-10 code consistent with end-stage kidney disease were excluded. Patients with an admission creatinine greater than or equal to 4 mg/dl were also excluded (Fig 1). Ethics approval was obtained from the institutional review board and the study operated under a waiver of informed consent from the Yale Human Research Protections Program.

### Study reporting

This study utilized the Strengthening the Reporting of Observation Studies in Epidemiology (STROBE) guidelines, as per the S1 Checklist, for reporting.

### Variable ascertainment

Demographics, vital signs, and laboratory data were obtained directly from the EHR, and all time-variable data (e.g., creatinine) was assigned a timestamp for further analyses. Our data set included the following variables, which were extracted electronically from the EHR: age, sex, race (measured as black or non-black), whether the admission was a surgical admission, patient history of congestive heart failure (CHF), hypertension, liver disease, on-admission Elixhauser score, laboratory findings including last bicarbonate, blood urea nitrogen (BUN), chloride, baseline creatinine, last creatinine, change in creatinine over the last 48 hours, hemoglobin, potassium, sodium, white blood cell count, platelet count, requirement during hospitalization for Bilevel Positive Airway Pressure (BiPAP), contrast studies, invasive ventilation, ICU admission, cardiac catheterization, red blood cell (RBC) transfusion, administration of angiotensin converting enzyme inhibitors (ACEs) or angiotensin II receptor blockers (ARBs), antibiotics, chemotherapy, diuretics, narcotics, nonsteroidal anti-inflammatory drugs (NSAIDs), vasopressors, proton pump inhibitors, and statins. Each patient would have a data row for each time point that a measurement (e.g., a vital sign) took place. Variables were carried forward through time until a new measurement for that variable took place. A visualization of this data set construction for a theoretical patient is provided in S1 Fig.

### Clinical definitions

AKI was defined per the Kidney Disease: Improving Global Outcomes (KDIGO) creatinine criteria [20]. Due to the relative sparsity of urine output data on the general hospital wards, urine output criteria for AKI were not considered. Thus, our definition of AKI was an increase in serum creatinine by 0.3 mg/dL in 48 hours or an increase in serum creatinine of 1.5 times baseline, which was defined as the lowest measured creatinine over the preceding 7 days. Also, given that the data set only contained inpatient data, community-acquired AKI was not studied in this model. The primary goal was to predict “AKI within 24 hours,” i.e., any set of measurements within 24 hours of AKI onset were considered “positive,” while any measurements prior to 24 hours of AKI onset (and all time points among the patients who did not develop AKI) were considered “negative.” AKI was only defined in relation to inpatient creatinine values; a patient’s “baseline” creatinine was not imputed from previous hospitalizations or outpatient lab studies. Time points before the first creatinine measurement would have “missing” values and AKI could not develop until a patient would have at least two measured creatinine values. Time points occurring after AKI onset were not included in modeling.

### Statistical methods

We used descriptive statistics to characterize patients across the three hospitals and by AKI status. To compare continuously and across the study hospitals, we used Kruskall–Wallis and chi-square testing, respectively. We assessed the univariable differences in patient characteristics at time points prior to AKI and not prior to AKI by univariable logistic regression with clustering at the patient level to account for the nonindependence of within-patient observations.

For our AKI prediction model, two-thirds of data from YNHH were used for training and one-third for internal validation. External validation was performed in the two other study hospitals (BH and SRH). We utilized a discrete-time logistic regression approach for prediction, with a new prediction being generated each time a covariate value was updated in the EHR. We again accounted for nonindependence of within-patient observations by clustering at the patient level. Candidate covariates were selected based on prior research and additionally considered variables measured in >90% of hospitalized patients (S1 Table) [21, 22]. Model covariates were divided into four classes: demographics, which included all time-invariant covariates; medications; and laboratory values and procedures. Given the uniquely strong performance of the rate of change of creatinine, we additionally modeled this feature independently. We assessed model performance using the AUC, again clustering at the patient level. The model was trained and evaluated on the primary outcome, a time-varying binary variable representing “AKI onset within 24 hours of this time point.” We measured covariate significance as the absolute value of the covariate’s Wald z-score in the full multivariable model.

While the primary outcome was AKI within 24 hours, we also evaluated model performance on the other kidney-relevant outcomes, including sustained AKI within 24 hours (defined as at least two consecutive creatinine values consistent with an AKI diagnosis), inpatient dialysis, and inpatient death.

We assumed time-varying variables were static after measurement, e.g., if the potassium level was measured as 5.1 meq/L, it was considered to be 5.1 meq/L at all future time points unless remeasured. Missing data was limited to laboratory values and represented less than 10% of all observations. Missing data were not imputed; it was decided to not generate predictions for time points when relevant data was unavailable, as this is what would occur in real-time should models be built into the medical record. In large data sets, case-wise deletion of incomplete records may be less biased than various imputation methods [23, 24]. Stata v. 15 (StataCorp., College Station, Texas) was used for all statistical tests.

### Ethics statement

Ethics approval was obtained from the institutional review board and the study operated under a waiver of informed consent from the Yale Human Research Protections Program.

## Results

After exclusion criteria were applied, the cohort contained 60,701 patients in the training set. The validation sets at YNHH, SRH, and BH contained 30,599 patients, 43,534 patients, and 35,025 patients, respectively. Table 1 displays baseline characteristics of patients included in the analysis. While significant differences existed across the hospitals, the cohort was characteristic of a hospitalized population with a median age ranging from 61 to 68 years. Statistically significant testing differences were expected between hospitals given very large sample sizes. Across hospitals, a range of 44%–49% of patients were male, 16%–20% were black, and 23%–29% were admitted to surgical wards.

**Table 1:** Baseline patient characteristics. Baseline patient characteristics in the training and validation sets. Data is in count (%) or median (IQR). There is significant difference across the data sets for all variables listed here at *P* < 0.001. Missing data is provided as a percentage of total time points included in the model (*N* = 22,743,165).

	YNHH Training Set (*N* = 60,701)	YNHH Validation Set (*N* = 30,599)	SRH (*N* = 43,534)	BH (*N* = 35,025)	Missing Data (%)
**Demographics**					
Age, y	61 (47–74)	60 (47–74)	68 (55–81)	65 (50–79)	0
Sex, male	29,649 (48.8%)	15,013 (49.1%)	19,478 (44.7%)	16,212 (46.3%)	0
Race, black	10,173 (16.8%)	5,066 (16.6%)	7,694 (17.7%)	6,973 (19.9%)	0
Surgical admission	17,265 (28.4%)	8,809 (28.8%)	11,197 (25.7%)	8,214 (23.5%)	0
**Laboratory**					
Bicarbonate, mmol/L	22.7 (20.6–24.9)	22.6 (20.5–24.8)	24 (22–26)	25 (23–28)	9.1
BUN, mg/dL	16 (11–24)	16 (11–24)	17 (12–25)	17 (12–25)	9.2
Chloride, mmol/L	102 (99–105)	102 (99–105)	102 (99–105)	103 (100–106)	9.8
Creatinine, mg/dL	0.9 (0.7–1.2)	0.9 (0.7–1.2)	0.9 (0.7–1.2)	0.9 (0.7–1.2)	8.9
Hemoglobin, g/dL	12.6 (10.8–14.1)	12.5 (10.8–14.1)	12.4 (10.9–13.8)	12.2 (10.6–13.6)	8.6
Platelets (x 10^9^/L)	232 (176–298)	232 (175–297)	227 (179–287)	224 (174–282)	8.7
Potassium, mmol/L	4 (3.7–4.4)	4 (3.7–4.4)	4.1 (3.7–4.4)	4.2 (3.9–4.6)	9.2
Sodium, mmol/L	138 (136–141)	138 (136–141)	139 (136–141)	139 (136–141)	9.1
White blood cells (cells/mm^3^)	9.1 (6.7–12.3)	9.1 (6.7–12.4)	9 (6.8–12.2)	9.1 (6.8–12.3)	8.7
**Medical History**					
CHF	9,038 (14.9%)	4,474 (14.6%)	7,806 (17.9%)	5,216 (14.9%)	0
Diabetes	13,124 (21.6%)	6,650 (21.7%)	10,599 (24.3%)	7,175 (20.5%)	0
Hypertension	5,369 (8.8%)	2,675 (8.7%)	4,989 (11.5%)	3,001 (8.6%)	0
Liver disease	4,556 (7.5%)	2,288 (7.5%)	2,348 (5.4%)	1,580 (4.5%)	0
Elixhauser score	3 (1–5)	3 (1–5)	3 (1–5)	2 (0–4)	0
**Medical Procedures**					
BiPAP use	1,991 (3.3%)	1,054 (3.4%)	1,999 (4.6%)	1,009 (2.9%)	0
Contrast study	14,267 (23.5%)	6,947 (22.7%)	6,177 (14.2%)	7,203 (20.6%)	0
Ventilation requirement	5,064 (8.3%)	2,473 (8.1%)	1,305 (3%)	1,642 (4.7%)	0
ICU	14,059 (23.2%)	6,911 (22.6%)	5,198 (11.9%)	4,429 (12.6%)	0
Cardiac catheterization	2,954 (4.9%)	1,548 (5.1%)	1,130 (2.6%)	1,106 (3.2%)	0
RBC transfusion	8,390 (13.8%)	4,128 (13.5%)	4,355 (10%)	3,099 (8.8%)	0
**Medications**					
ACE or ARB	12,427 (20.5%)	6,298 (20.6%)	11,699 (26.9%)	8,452 (24.1%)	0
Antibiotic	37,041 (61%)	18,558 (60.6%)	29,321 (67.4%)	17,227 (49.2%)	0
Chemotherapy	1,481 (2.4%)	708 (2.3%)	160 (0.4%)	312 (0.9%)	0
Diuretic	16,707 (27.5%)	8,467 (27.7%)	13,949 (32%)	8,373 (23.9%)	0
Narcotic	38,046 (62.7%)	19,152 (62.6%)	26,506 (60.9%)	16,487 (47.1%)	0
NSAID	5,928 (9.8%)	3,020 (9.9%)	6,678 (15.3%)	3,407 (9.7%)	0
Vasopressor	11,091 (18.3%)	5,497 (18%)	7,955 (18.3%)	3,798 (10.8%)	0
Proton pump inhibitor	23,967 (39.5%)	11,852 (38.7%)	20,421 (46.9%)	11,864 (33.9%)	0
Statin	11,637 (19.2%)	5,854 (19.1%)	10,250 (23.5%)	5,967 (17.0%)	0

**Abbreviations**: ACE, angiotensin converting enzyme inhibitor; ARB, angiotensin II receptor blockers; AKI, acute kidney injury; BH, Bridgeport Hospital; BiPAP, Bilevel Positive Airway Pressure; BUN, blood urea nitrogen; CHF, congestive heart failure; ICU, intensive care unit; IQR, interquartile range; NSAID, nonsteroidal anti-inflammatory drug; SRH, Saint Raphael's Hospital; RBC, red blood cell; YNHH, Yale New Haven Hospital.

Outcomes across the three study hospitals appear in Table 2. There was substantial heterogeneity in the rates of the primary outcome, with AKI rates ranging from 11.4%–19.1% between hospitals. Similar heterogeneity was noted between study hospitals when data was stratified by AKI stage.

**Table 2:** Patient outcomes within training and testing cohort. Outcomes within the training and test cohort. Values are *N* (%). *P* values represent differences across the three test cohorts.

	YNHH Training Set	YNHH Validation Set	SRH	BH	*P* value
N	60,701	30,599	43,534	35,025	<0.001
AKI (any stage)	11,593 (19.1)	5,772 (18.9)	7,416 (17.0)	3,977 (11.4)	<0.001
AKI Stage 1	9,366 (15.4)	4,657 (15.2)	6,194 (14.2)	3,157 (9.0)	<0.001
AKI Stage 2	1,578 (2.6)	758 (2.5)	916 (2.1)	542 (1.5)	<0.001
AKI Stage 3	657 (1.1)	364 (1.2)	308 (0.7)	295 (0.8)	<0.001
Sustained AKI	6,196 (10.2)	3,056 (10.0)	3,463 (8.0)	2,070 (5.9)	<0.001
Renal replacement therapy	341 (0.6)	204 (0.7)	117 (0.3)	135 (0.4)	<0.001
Death	1,698 (2.8)	868 (2.8)	696 (1.6)	760 (2.2)	<0.001

**Abbreviations:** AKI, acute kidney injury; BH, Bridgeport Hospital; SRH, Saint Raphael's Hospital; YNHH, Yale New Haven Hospital.

Within the training set, there were 8,302,779 time points assessed during time periods not associated with AKI in the following 24 hours and 459,456 time points during time periods within 24 hours of AKI onset. The median (interquartile range [IQR]) number of predictions generated per patient in the training set was 99 (61–169). Table 3 characterizes the time-invariant and time-variant data at those time points. In univariable analysis, those who developed AKI tended to be older, male, and black. All medical comorbidities evaluated, namely CHF, diabetes, hypertension, and liver disease, were significantly more prevalent in patients who developed AKI. With respect to laboratory studies, patients who would imminently develop AKI had higher BUN and creatinine. Initiation of BiPAP, mechanical ventilation, transfer to the ICU, cardiac catheterization, and RBC transfusion were all more prevalent in patients with imminent AKI. Receiving a contrast study was inversely correlated with imminent AKI. With respect to medications, chemotherapeutics and NSAID exposure were inversely correlated to AKI development, whereas antibiotics, diuretics, narcotics, vasopressors, ACE/ARBs, proton pump inhibitors, and statins were associated with increased risk. Multivariable model odds ratios appear in S1 Table.

**Table 3:** Characteristics of patients at times not prior to AKI versus prior to AKI. Characteristics and univariable comparisons between times within 24 hours of AKI onset versus times not prior to AKI onset in the training set. Data is count (%) or median (IQR).

	No AKI in 24 (*N* = 8,302,779)	Yes AKI in 24 (*N* = 459,456)	*P* value
**Demographics**			
Age, y	61 (48–73)	66 (53–77)	<0.001
Sex, male	50.2%	54.4%	<0.001
Race, black	15.1%	16.2%	0.006
Surgical admission	21.4%	24.6%	<0.001
**Laboratory**			
Bicarbonate, mmol/L	22.8 (20.8–25)	22 (19.5–24.4)	<0.001
BUN, mg/dL	15 (10–22)	19 (13–29)	<0.001
Chloride, mmol/L	103 (100–105)	103 (99–106)	0.14
Creatinine, mg/dL	0.8 (0.6–1)	1 (0.7–1.4)	<0.001
Change in creatinine over last 48 hours, mg/dL	0 (0–0)	0 (0–0.1)	<0.001
Hemoglobin, g/dL	11.2 (9.5–12.9)	11 (9.4–12.7)	<0.001
Platelets (x 10^9^/L)	221 (159–295)	203 (141–277)	<0.001
Potassium, mmol/L	4 (3.7–4.3)	4.1 (3.7–4.4)	<0.001
Sodium, mmol/L	138 (136–141)	138 (136–141)	0.08
White blood cells (cells/mm3)	8.7 (6.3–11.8)	9.9 (7–13.6)	<0.001
**Medical History**			
CHF	15.9%	30.7%	<0.001
Diabetes	21.8%	28.9%	<0.001
Hypertension	7.8%	18.5%	<0.001
Liver disease	7.9%	11.9%	<0.001
Elixhauser score	3 (1–5)	4 (3–6)	<0.001
**Medical Procedures**			
BiPAP use	4.4%	7.6%	<0.001
Contrast study	27.6%	25.4%	<0.001
Ventilation requirement	13.8%	28.4%	<0.001
ICU	20.2%	40%	<0.001
Cardiac catheterization	4.1%	8%	<0.001
RBC transfusion	18.1%	25.1%	<0.001
**Medications**			
ACE or ARB	13.9%	14.7%	0.02
Antibiotic	63.5%	68.8%	<0.001
Chemotherapy	2.7%	1.6%	<0.001
Diuretic	23.6%	36.1%	<0.001
Narcotic	64.6%	68.5%	<0.001
NSAID	5.8%	3.9%	<0.001
Vasopressor	22.2%	33.9%	<0.001
Proton pump inhibitor	36.7%	38.1%	0.007
Statin	14.7%	15.5%	0.03

**Abbreviations**: ACE, angiotensin converting enzyme inhibitor; ARB, angiotensin II receptor blockers; AKI, acute kidney injury; BiPAP, Bilevel Positive Airway Pressure; BUN, blood urea nitrogen; CHF, congestive heart failure; ICU, intensive care unit; IQR, interquartile range; NSAID, nonsteroidal anti-inflammatory drug; RBC, red blood cell.

**Fig 2:**
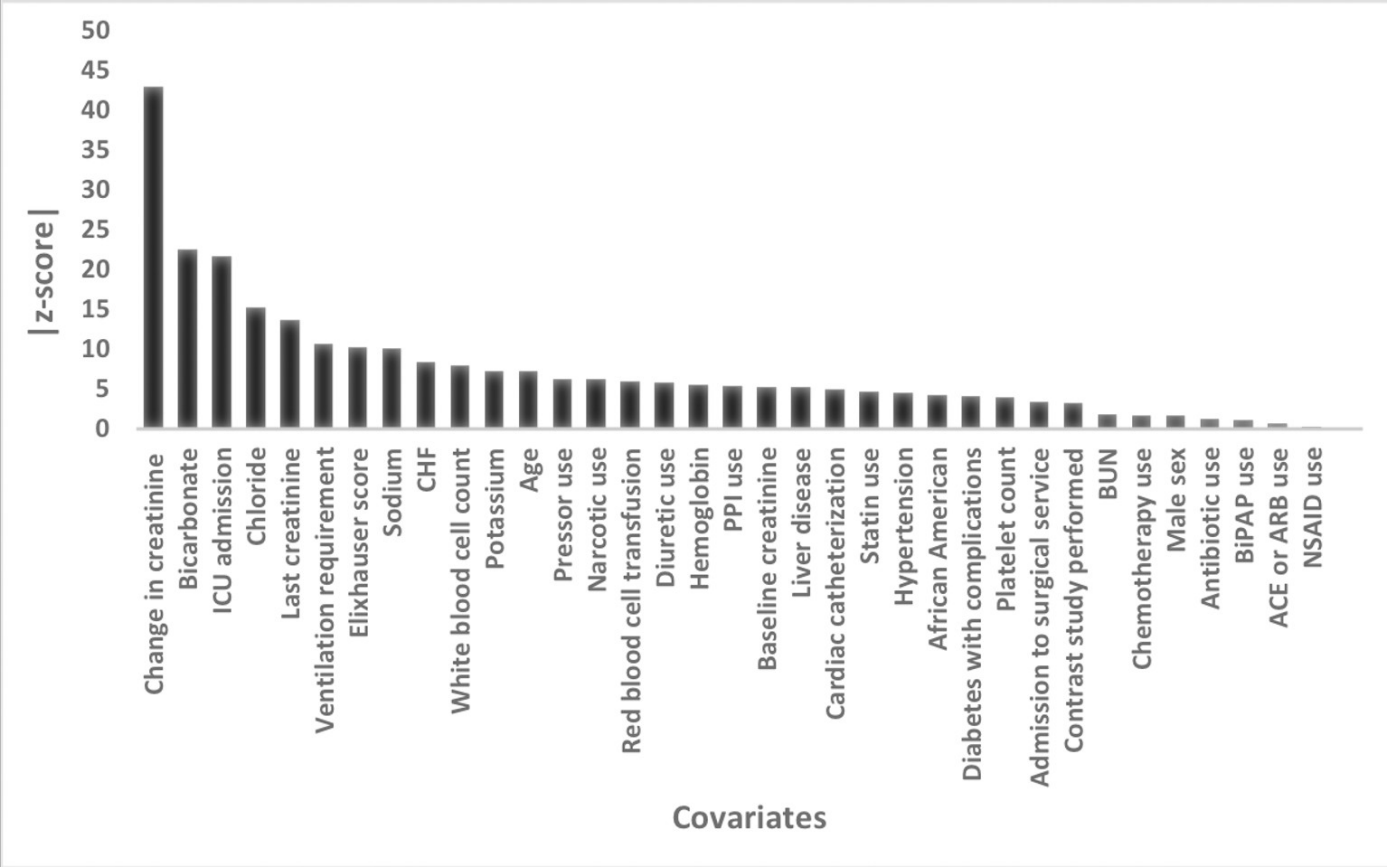
Performance of model covariates within the fully adjusted model. Higher absolute value of Wald z-scores indicate a greater degree of statistical significance within the predictive model.

Fig 2 displays the contribution of the various covariates for AKI prediction in the full multivariable model. Change in creatinine over the past 48 hours was by far the strongest predictor of imminent AKI; however, several other variables were strongly predictive, including admission to the ICU, most recent creatinine, requirement for ventilation, Elixhauser score, serum sodium, bicarbonate, and chloride concentration. After multivariable adjustment, the protective effect of NSAIDs and chemotherapy exposure was no longer seen.

**Fig 3:**
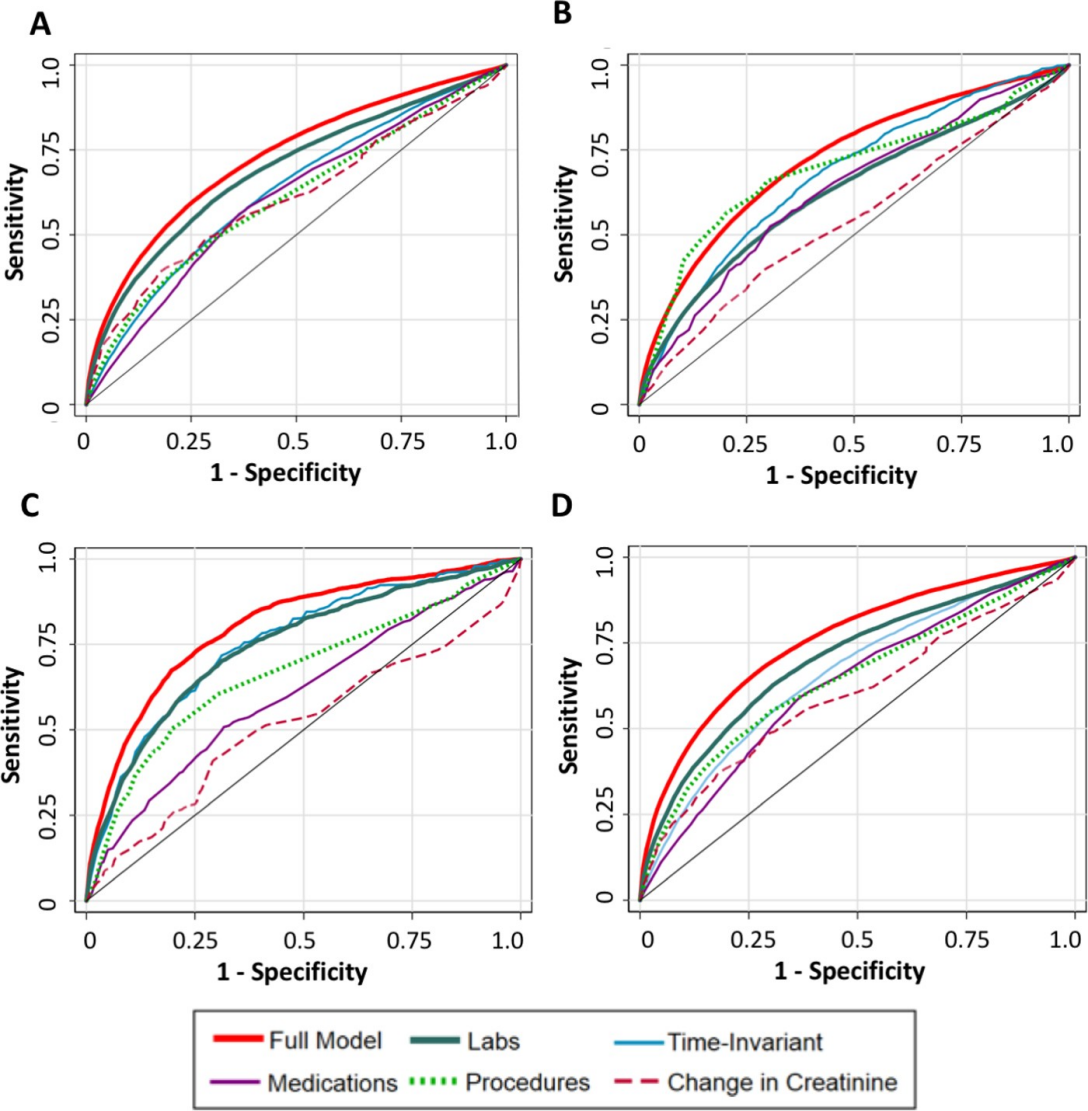
ROC curves of the various AKI models. Curves reflect performance in a test set composed of a combination of the internal and external validation cohorts. (A) Prediction of AKI in 24 hours. (B) Prediction of hospital mortality. (C) Prediction of need for renal replacement therapy. (D) Prediction of sustained AKI. AKI, acute kidney injury; ROC, receiver-operator characteristic.

Model performance in terms of prediction of imminent AKI, imminent sustained AKI, renal replacement therapy, and death is reported in Table 4. The full model, which includes time-invariant, medication, laboratory study, and procedure data, performed the best of all models evaluated (average AUC across hospitals of 0.73) in terms of imminent prediction of AKI. Among the simpler models, models utilizing time-updated laboratory values performed best (average AUC 0.69). The complete model similarly performed strongest for prediction of other clinically relevant outcomes, such as renal replacement therapy (average AUC 0.82), sustained AKI (average AUC 0.76), and mortality (average AUC 0.72). Again, the model containing only time-updated laboratory values had similar performance in predicting all of these outcomes. Model performance was generally stable in all models for predictions on the validation data set and two external data sets. AUC curves for the various models and outcomes are displayed in Fig 3. The closed form equation of the laboratory-value model along with coefficients is provided in S2 Fig.

**Table 4:** Model performance for prediction of 24-hour AKI and related outcomes. Performance of multivariable models for prediction of 24-hour AKI, sustained AKI, renal replacement therapy, and inpatient mortality, with columns signifying models utilizing different subsets of input variables. Model performance is displayed as AUC for each model when applied to data from the YNHH validation set and SRH and BH data sets.

	AUC (95% CI)					
	Full Model	Demographic	Medications	Change in Creatinine	Procedures	Laboratory Studies
	**24-hour AKI**					
YNHH validation set	0.74 (0.73–0.74)	0.65 (0.64–0.66)	0.59 (0.59–0.60)	0.61 (0.60–0.61)	0.63 (0.62–0.63)	0.69 (0.68–0.70)
SRH	0.69 (0.68–0.69)	0.62 (0.61–0.63)	0.60 (0.59–0.61)	0.59 (0.58–0.60)	0.59 (0.58–0.59)	0.64 (0.63–0.65)
BH	0.76 (0.75–0.77)	0.63 (0.62–0.65)	0.62 (0.61–0.63)	0.64 (0.63–0.65)	0.60 (0.59–0.61)	0.74 (0.73–0.75)
	**Sustained AKI**					
YNHH validation set	0.77 (0.76–0.78)	0.68 (0.67–0.69)	0.62 (0.61–0.63)	0.60 (0.59–0.61)	0.66 (0.65–0.67)	0.70 (0.69–0.72)
SRH	0.72 (0.71–0.73)	0.65 (0.64–0.66)	0.62 (0.61–0.63)	0.57 (0.56–0.58)	0.62 (0.61–0.63)	0.65 (0.64–0.66)
BH	0.79 (0.78–0.80)	0.65 (0.64–0.67)	0.63 (0.62–0.65)	0.63 (0.62–0.65)	0.63 (0.61–0.64)	0.76 (0.74–0.77)
	**Renal Replacement Therapy**					
YNHH validation set	0.79 (0.73–0.85)	0.79 (0.73–0.85)	0.60 (0.52–0.67)	0.51 (0.48–0.54)	0.67 (0.61–0.74)	0.73 (0.66–0.79)
SRH	0.85 (0.80–0.89)	0.78 (0.73–0.83)	0.61 (0.55–0.67)	0.51 (0.46–0.57)	0.62 (0.55–0.69)	0.83 (0.78–0.88)
BH	0.78 (0.74–0.82)	0.72 (0.66–0.78)	0.56 (0.49–0.63)	0.50 (0.47–0.54)	0.62 (0.57–0.68)	0.70 (0.64–0.75)
	**Inpatient Mortality**					
YNHH validation set	0.69 (0.67–0.72)	0.66 (0.64–0.69)	0.59 (0.56–0.62)	0.53 (0.51–0.54)	0.67 (0.65–0.70)	0.60 (0.57–0.63)
SRH	0.75 (0.73–0.77)	0.74 (0.71–0.76)	0.67 (0.64–0.69)	0.53 (0.52–0.55)	0.72 (0.69–0.75)	0.63 (0.60–0.66)
BH	0.73 (0.71–0.75)	0.68 (0.65–0.71)	0.63 (0.60–0.66)	0.56 (0.55–0.58)	0.66 (0.63–0.70)	0.65 (0.62–0.67)

**Abbreviations:** AKI, acute kidney injury; AUC, area under the ROC curve; BH, Bridgeport Hospital; SRH, Saint Raphael's Hospital; YNHH, Yale New Haven Hospital.

## Discussion

In this study, we assessed the performance of a predictive model built from EHR data from three US hospitals to predict the onset of AKI within 24 hours. Our complete model, which utilized all potential covariates, displayed moderately good performance for predicting 24-hour AKI (average AUC across hospitals of 0.73) as well as the clinically pertinent outcomes of requirement for renal replacement therapy and mortality. A simpler model utilizing only time-updated laboratory data performed nearly as well as the complete model and maintained its performance across the three hospitals and across the outcomes of sustained AKI and requirement for renal replacement therapy.

Several variables revealed themselves as having strong relationships with respect to impending AKI. Change in creatinine over the last 48 hours was by far the most predictive variable; however, change in creatinine alone had poor predictive ability across all outcomes. As was expected, patients sicker at baseline and those with higher hospitalization acuity were at higher risk for developing AKI. Specifically, patients with CHF and liver disease were at higher risk, as were patients who required ICU admission, ventilation, vasopressors, or cardiac catheterization. Several variables typically tied to renal injury, namely NSAID use, ACE/ARB use, and contrast studies contributed minimally to imminent AKI prediction in the multivariable models. We hypothesize providers cautiously order these medications and studies for inpatients, and these unmeasured nuances of provider behavior limit the utility of these variables for AKI prediction. Such selection bias has been previously discussed regarding studying the relationship of contrast studies and AKI and may reflect a similar phenomenon with NSAIDs and ACE/ARBs [25].

Prior efforts have shown that electronic alerts have seen mixed results; some studies show no benefit, whereas others show benefit for AKI progression when tied to nephrology consultation or other interventions [21, 26, 27]. One recent study developed an e-alert prediction tool for hospital-acquired AKI, which showed improved outcomes [28]. AUC for this tool, however, was inferior to our simple laboratory model, and the variables used may be difficult to operationalize, as they combine chart-documented medical history as well as physical exam findings [29]. Our model, while maintaining similar predictive performance to previously published models, uses only commonly measured laboratory data for making its predictions. By avoiding variables such as nursing and provider documentation, subjective patient assessments, and institution-specific hospital events, we suggest that our model may be implemented more easily than preceding models onto other EHRs than preceding models without a loss of predictive performance.

### Strengths

Our study has several strengths. First, our model is generalizable to a variety of inpatient care settings, as it was developed on a large cohort from a tertiary care center (YNHH) and further validated at a community teaching (SRH) and nonteaching (BH) institution and maintained good predictive performance across the three diverse care settings. Second, our model is generalizable to a variety of inpatients, as it was developed on data from patients in both hospital floor, surgical, and ICU settings. Lastly, our laboratory-based model is simple and holds promise for ready implementation into the EHR; this model is currently live and being evaluated prospectively in one of the study hospitals.

### Limitations

Our study should be interpreted in the light of several limitations. First, the predictability of the model yielded similar AUCs to prior models with respect to predicting at least stage I AKI; thus, we do not claim to have developed a model that is far superior to other models [10, 16, 17]. We also recognize that a recent study found AUCs of 0.9 for AKI prediction; we note, however, that this model predicted stage II AKI (or greater) rather than stage I (or greater), and our goal was to predict all hospital AKI irrespective of severity [16]. We hypothesize preventing stage I may be more clinically beneficial for reversing disease rather than a stage II prediction, at which point it may be too late to change disease course. Additionally, this and other models contain a high quantity of variables, some calculated, which may be difficult to operationalize across diverse health systems and EHR platforms. Second, our model is descriptive in nature; while it can reveal factors associated with imminent AKI, it is unable to imply causality or provide explanations for unexpected findings. Third, given sparsity of data, we were unable to include urine output into the model, and we recognize that urine output has significant implications in terms of AKI incidence [30]. In addition, because we limit our definition of AKI to look back 7 days from admission for baseline creatinine, it is likely that our model misses some patients who present with AKI but do not have recent creatinine values from which the patient would meet our definition. Fourth, all hospitals included in our analysis utilized the Epic EHR system, and the model’s performance should ultimately be validated on healthcare data obtained from other EHRs. A final limitation is that our model does not suggest the best manner in which providers should react to notification of imminent AKI in a patient.

### Future implications

To date, there remain limited interventions for treatment of hospitalized patients who develop AKI. The development of predictive models could aid in AKI prevention and change a patient’s course of disease.

The parsimonious model developed in this study should be further evaluated on other large hospital data sets to validate model performance. Further work should be performed to determine optimal implementation of such a model into the EHR and how best to use the predictions to affect provider behaviors while minimizing intrusiveness of such interventions and maintaining sensitivity to increasing concerns of “alert fatigue” [31].

The laboratory model presented in this paper can be implemented in practice with different cut-points for prediction to either optimize sensitivity or specificity of a model. For example, from our AUROC curves of the laboratory-only model, prediction could be generated with approximately 80% sensitivity and 50% specificity. With a prevalence of AKI of approximately 15% (as seen in our data set), such a prediction would carry a positive predictive value (PPV) of 22%. This cut-point may be well-suited for low-level interventions (such as additional monitoring or avoidance of nephrotoxins). Alternatively, a cut point could be generated with approximately 87.5% specificity but only 37.5% sensitivity. At the same prevalence, this prediction would be expected to have a PPV of 35%. With this more specific prediction, one might consider providing higher-level interventions, such as provision of intravenous fluids or usage of pharmacy consultants. Further work needs to study such model implementations and see how they best fit within workflow and optimize patient care.

In addition, after the deployment of such a model on the EHR, specific interventions could be randomized and tested among patients to evaluate novel therapies for prevention of AKI.

### Conclusion

Our study developed an original model for 24-hour prediction of AKI in hospitalized patients with good predictability in the domains of AKI prediction as well as sustained AKI, requirement for renal replacement therapy, and mortality. Previously, integration of real-time predictive analytics for AKI have been limited, in part due to implementation difficulties and in part due to large model complexity [32–34]. Compared to prior studies, our study has shown that a parsimonious model using only laboratory values maintains good performance and lends itself to ready implementation in EHR. We anticipate that broad implementation of this algorithm would change the treatment paradigm of AKI from reactive to proactive, which will afford new opportunities for the evaluation, diagnosis, and treatment of the syndrome.

## Supporting information

S1 Checklist (PDF)

S1 Fig (TIF)

S2 Fig (TIF)

S1 Table (XLSX)

## Abbreviations

ACE: angiotensin converting enzyme inhibitor
AKI: acute kidney injury
ARB: angiotensin II receptor blocker
AUC: area under the curve
BH: Bridgeport Hospital
BiPAP: Bilevel Positive Airway Pressure
BUN: blood urea nitrogen
CHF: congestive heart failure
EHR: electronic health record
ICU: intensive care unit
IQR: interquartile range
KDIGO: Kidney Disease: Improving Global Outcomes
NSAID: nonsteroidal anti-inflammatory drug
PPV: positive predictive value
SRH: Saint Raphael’s Hospital
RBC: red blood cell
ROC: receiver-operator characteristic
YNHH: Yale New Haven Hospital

## References

[1] Chertow GM, Burdick E, Honour M, Bonventre JV, Bates DW. Acute kidney injury, mortality, length of stay, and costs in hospitalized patients. J Am Soc Nephrol. 2005;16(11):3365–70. 10.1681/ASN.2004090740 . 16177006

[2] Zeng X, McMahon GM, Brunelli SM, Bates DW, Waikar SS. Incidence, outcomes, and comparisons across definitions of AKI in hospitalized individuals. Clin J Am Soc Nephrol. 2014;9(1):12–20. 10.2215/CJN.02730313 ; PubMed Central PMCID: 3878695. 24178971 PMC3878695

[3] CfMaM S. 2018. Available from: https://www.cms.gov/Medicare/Quality-Initiatives-Patient-Assessment-Instruments/MMS/PC-Updates-on-Previous-Comment-Periods.html. Accessed January 1, 2019.

[4] Moran SM, Myers BD. Course of acute renal failure studied by a model of creatinine kinetics. Kidney Int. 1985;27(6):928–37. . 4021321 10.1038/ki.1985.101

[5] Edelstein CL. Biomarkers of acute kidney injury. Adv Chronic Kidney Dis. 2008;15(3):222–34. 10.1053/j.ackd.2008.04.003 ; PubMed Central PMCID: 3287955. 18565474 PMC3287955

[6] de Geus HR, Betjes MG, Bakker J. Biomarkers for the prediction of acute kidney injury: a narrative review on current status and future challenges. Clin Kidney J. 2012;5(2):102–8. 10.1093/ckj/sfs008 ; PubMed Central PMCID: 3341843. 22833807 PMC3341843

[7] Raghupathi W, Raghupathi V. Big data analytics in healthcare: promise and potential. Health Inf Sci Syst. 2014;2:3 10.1186/2047-2501-2-3 ; PubMed Central PMCID: 4341817. 25825667 PMC4341817

[8] Obermeyer Z, Emanuel EJ. Predicting the Future—Big Data, Machine Learning, and Clinical Medicine. N Engl J Med. 2016;375(13):1216–9. 10.1056/NEJMp1606181 ; PubMed Central PMCID: 5070532. 27682033 PMC5070532

[9] Kate RJ, Perez RM, Mazumdar D, Pasupathy KS, Nilakantan V. Prediction and detection models for acute kidney injury in hospitalized older adults. BMC Med Inform Decis Mak. 2016;16:39 10.1186/s12911-016-0277-4 ; PubMed Central PMCID: 4812614. 27025458 PMC4812614

[10] Koyner JL, Adhikari R, Edelson DP, Churpek MM. Development of a Multicenter Ward-Based AKI Prediction Model. Clin J Am Soc Nephrol. 2016;11(11):1935–43. 10.2215/CJN.00280116 ; PubMed Central PMCID: 5108182. 27633727 PMC5108182

[11] Gurm HS, Seth M, Kooiman J, Share D. A novel tool for reliable and accurate prediction of renal complications in patients undergoing percutaneous coronary intervention. J Am Coll Cardiol. 2013;61(22):2242–8. 10.1016/j.jacc.2013.03.026 . 23721921

[12] Wijeysundera DN, Karkouti K, Dupuis JY, Rao V, Chan CT, Granton JT, et al Derivation and validation of a simplified predictive index for renal replacement therapy after cardiac surgery. JAMA. 2007;297(16):1801–9. 10.1001/jama.297.16.1801 . 17456822

[13] Laszczynska O, Severo M, Azevedo A. Electronic Medical Record-Based Predictive Model for Acute Kidney Injury in an Acute Care Hospital. Stud Health Technol Inform. 2016;228:810–2. . 27577501

[14] Kane-Gill SL, Sileanu FE, Murugan R, Trietley GS, Handler SM, Kellum JA. Risk factors for acute kidney injury in older adults with critical illness: a retrospective cohort study. Am J Kidney Dis. 2015;65(6):860–9. 10.1053/j.ajkd.2014.10.018 ; PubMed Central PMCID: 4442750. 25488106 PMC4442750

[15] Sanchez-Pinto LN, Khemani RG. Development of a Prediction Model of Early Acute Kidney Injury in Critically Ill Children Using Electronic Health Record Data. Pediatr Crit Care Med. 2016;17(6):508–15. 10.1097/PCC.0000000000000750 . 27124567

[16] Koyner JL, Carey KA, Edelson DP, Churpek MM. The Development of a Machine Learning Inpatient Acute Kidney Injury Prediction Model. Crit Care Med. 2018;46(7):1070–7. 10.1097/CCM.0000000000003123 . 29596073

[17] Mohamadlou H, Lynn-Palevsky A, Barton C, Chettipally U, Shieh L, Calvert J, et al Prediction of Acute Kidney Injury With a Machine Learning Algorithm Using Electronic Health Record Data. Can J Kidney Health Dis. 2018;5:2054358118776326. Epub 2018/08/11. 10.1177/2054358118776326 30094049 PMC6080076

[18] Haase M, Bellomo R, Devarajan P, Ma Q, Bennett MR, Mockel M, et al Novel biomarkers early predict the severity of acute kidney injury after cardiac surgery in adults. Ann Thorac Surg. 2009;88(1):124–30. 10.1016/j.athoracsur.2009.04.023 . 19559209

[19] Huang C, Murugiah K, Mahajan S, Li SX, Dhruva SS, Haimovich JS, et al Enhancing the prediction of acute kidney injury risk after percutaneous coronary intervention using machine learning techniques: A retrospective cohort study. PLoS Med. 2018;15(11):e1002703 Epub 2018/11/28. 10.1371/journal.pmed.1002703 . 30481186 PMC6258473

[20] Summary of Recommendation Statements. Kidney Int Suppl (2011). 2012;2(1):8–12. 10.1038/kisup.2012.7 ; PubMed Central PMCID: 4089654. 25018916 PMC4089654

[21] Wilson FP, Shashaty M, Testani J, Aqeel I, Borovskiy Y, Ellenberg SS, et al Automated, electronic alerts for acute kidney injury: a single-blind, parallel-group, randomised controlled trial. Lancet. 2015;385(9981):1966–74. 10.1016/S0140-6736(15)60266-5 ; PubMed Central PMCID: 4475457. 25726515 PMC4475457

[22] Koyner JL, Garg AX, Thiessen-Philbrook H, Coca SG, Cantley LG, Peixoto A, et al Adjudication of etiology of acute kidney injury: experience from the TRIBE-AKI multi-center study. BMC Nephrol. 2014;15:105 Epub 2014/07/06. 10.1186/1471-2369-15-105 24996668 PMC4091753

[23] Kang H. The prevention and handling of the missing data. Korean J Anesthesiol. 2013;64(5):402–6. 10.4097/kjae.2013.64.5.402 ; PubMed Central PMCID: 3668100. 23741561 PMC3668100

[24] Sterne JA, White IR, Carlin JB, Spratt M, Royston P, Kenward MG, et al Multiple imputation for missing data in epidemiological and clinical research: potential and pitfalls. BMJ. 2009;338:b2393 10.1136/bmj.b2393 ; PubMed Central PMCID: 2714692. 19564179 PMC2714692

[25] Vandenberghe W, De Corte W, Hoste EA. Contrast-associated AKI in the critically ill: relevant or irrelevant? Curr Opin Crit Care. 2014;20(6):596–605. Epub 2014/10/15. 10.1097/MCC.0000000000000156 . 25314241

[26] Park S, Baek SH, Ahn S, Lee KH, Hwang H, Ryu J, et al Impact of Electronic Acute Kidney Injury (AKI) Alerts With Automated Nephrologist Consultation on Detection and Severity of AKI: A Quality Improvement Study. Am J Kidney Dis. 2018;71(1):9–19. 10.1053/j.ajkd.2017.06.008 . 28754457

[27] Kolhe NV, Reilly T, Leung J, Fluck RJ, Swinscoe KE, Selby NM, et al A simple care bundle for use in acute kidney injury: a propensity score-matched cohort study. Nephrol Dial Transplant. 2016;31(11):1846–54. 10.1093/ndt/gfw087 . 27190331

[28] Hodgson LE, Roderick PJ, Venn RM, Yao GL, Dimitrov BD, Forni LG. The ICE-AKI study: Impact analysis of a Clinical prediction rule and Electronic AKI alert in general medical patients. PLoS ONE. 2018;13(8):e0200584 10.1371/journal.pone.0200584 ; PubMed Central PMCID: 6082509. 30089118 PMC6082509

[29] Hodgson LE, Dimitrov BD, Roderick PJ, Venn R, Forni LG. Predicting AKI in emergency admissions: an external validation study of the acute kidney injury prediction score (APS). BMJ Open. 2017;7(3):e013511 Epub 2017/03/10. 10.1136/bmjopen-2016-013511 28274964 PMC5353262

[30] Quan S, Pannu N, Wilson T, Ball C, Tan Z, Tonelli M, et al Prognostic implications of adding urine output to serum creatinine measurements for staging of acute kidney injury after major surgery: a cohort study. Nephrol Dial Transplant. 2016;31(12):2049–56. 10.1093/ndt/gfw374 . 27941063

[31] Ancker JS, Edwards A, Nosal S, Hauser D, Mauer E, Kaushal R, et al Effects of workload, work complexity, and repeated alerts on alert fatigue in a clinical decision support system. BMC Med Inform Decis Mak. 2017;17(1):36 Epub 2017/04/12. 10.1186/s12911-017-0430-8 28395667 PMC5387195

[32] Goldstein BA, Navar AM, Pencina MJ, Ioannidis JP. Opportunities and challenges in developing risk prediction models with electronic health records data: a systematic review. J Am Med Inform Assoc. 2017;24(1):198–208. 10.1093/jamia/ocw042 ; PubMed Central PMCID: 5201180. 27189013 PMC5201180

[33] Wu J, Roy J, Stewart WF. Prediction modeling using EHR data: challenges, strategies, and a comparison of machine learning approaches. Med Care. 2010;48(6 Suppl):S106–13. 10.1097/MLR.0b013e3181de9e17 . 20473190

[34] Bates DW, Saria S, Ohno-Machado L, Shah A, Escobar G. Big data in health care: using analytics to identify and manage high-risk and high-cost patients. Health Aff (Millwood). 2014;33(7):1123–31. 10.1377/hlthaff.2014.0041 . 25006137

