# Supplementary figures and images for: A simple real-time model for predicting acute kidney injury in hospitalized patients in the US: A descriptive modeling study
Source: PLoS Med. 2019 Jul 15;16(7):e1002861. doi: 10.1371/journal.pmed.1002861 (PMC6629054; doi:10.1371/journal.pmed.1002861)

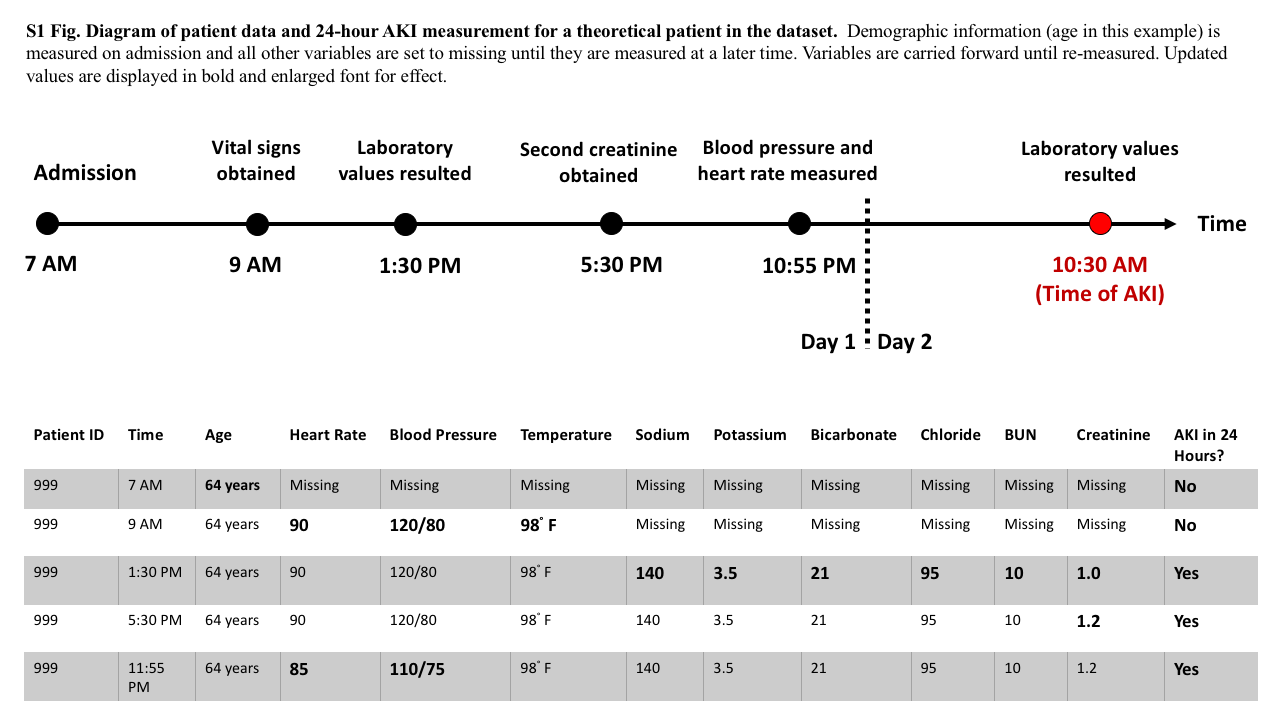

Supplement: S1 Fig — (TIF) [file pmed.1002861.s002.tif]

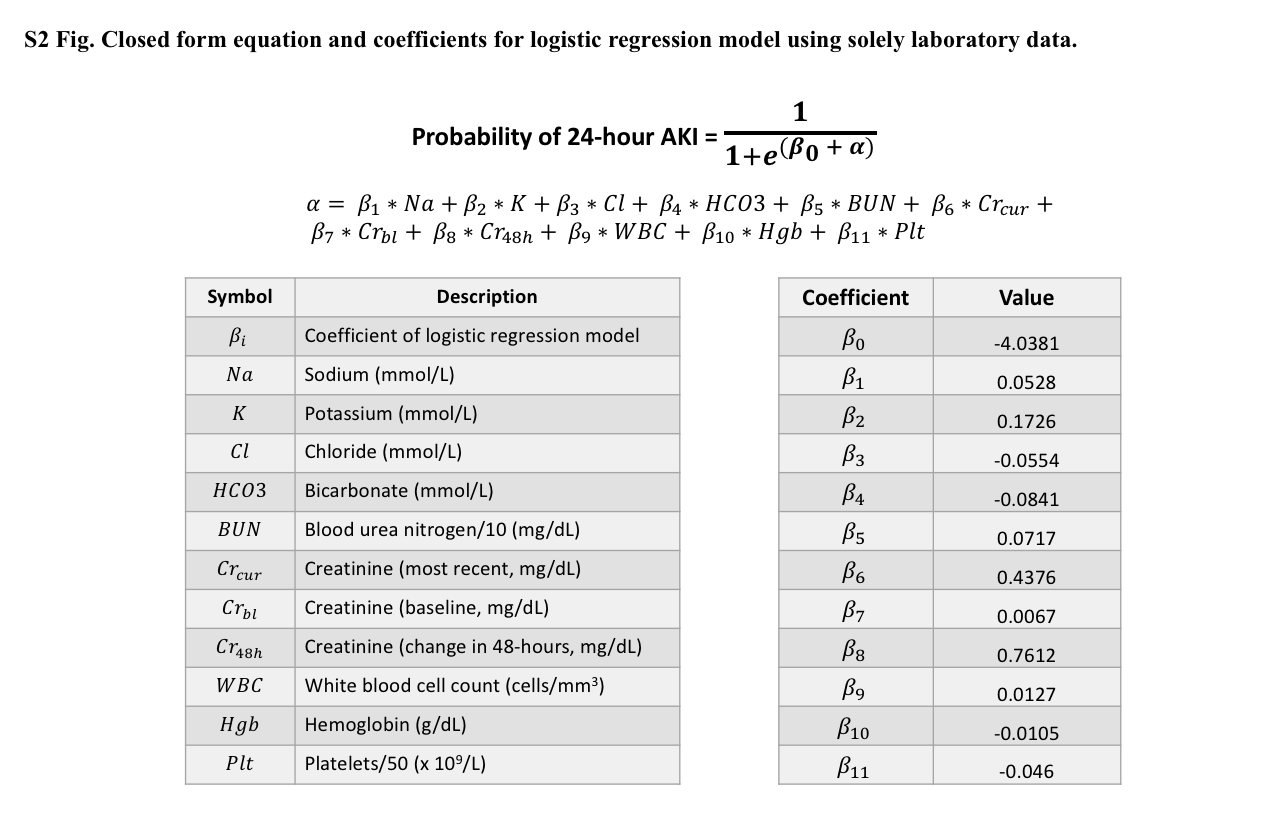

Supplement: S2 Fig — (TIF) [file pmed.1002861.s003.tif]
